# Fake anti-malarials: start with the facts

**DOI:** 10.1186/s12936-016-1096-x

**Published:** 2016-02-13

**Authors:** Harparkash Kaur, Siȃn Clarke, Mirza Lalani, Souly Phanouvong, Philippe Guérin, Andrew McLoughlin, Benjamin K. Wilson, Michael Deats, Aline Plançon, Heidi Hopkins, Debora Miranda, David Schellenberg

**Affiliations:** London School of Hygiene and Tropical Medicine, London, WC1E 7HT UK; Promoting the Quality of Medicines Program, U.S. Pharmacopeial Convention, Rockville, MD USA; Worldwide Antimalarial Resistance Network (WWARN), University of Oxford, Oxford, UK; Centre for Tropical Medicine and Global Health, Nuffield Department of Clinical Medicine, University of Oxford, Oxford, UK; The Global Fund to Fight Aids, Tuberculosis and Malaria, Geneva, Switzerland; Intellectual Ventures Laboratory, Bellevue, WA USA; SSFFC Surveillance and Monitoring, Safety and Vigilance, Essential Medicines and Health Products, World Health Organization, Geneva, Switzerland; Medical Product Counterfeiting and Pharmaceutical Crime Sub-Directorate, INTERPOL, Lyon, France

**Keywords:** Drug quality, Falsified, Substandard, Artemisinin combination therapy, Anti-malarials, Degraded drugs

## Abstract

This meeting report presents the key findings and discussion points of a 1-day meeting entitled ‘Fake anti-malarials: start with the facts’ held on 28th May 2015, in Geneva, Switzerland, to disseminate the findings of the artemisinin combination therapy consortium’s drug quality programme. The teams purchased over 10,000 samples, using representative sampling approaches, from six malaria endemic countries: Equatorial Guinea (Bioko Island), Cambodia, Ghana, Nigeria, Rwanda and Tanzania. Laboratory analyses of these samples showed that falsified anti-malarials (<8 %) were found in just two of the countries, whilst substandard artemisinin-based combinations were present in all six countries and, artemisinin-based monotherapy tablets are still available in some places despite the fact that the WHO has urged regulatory authorities in malaria-endemic countries to take measures to halt the production and marketing of these oral monotherapies since 2007. This report summarizes the presentations that reviewed the public health impact of falsified and substandard drugs, sampling strategies, techniques for drug quality analysis, approaches to strengthen health systems capacity for the surveillance of drug quality, and the ensuing discussion points from the dissemination meeting.

## Background

Artemisinin-based combination therapy (ACT) is recommended as first line treatment for malaria treatment by the World Health Organization (WHO) and effective malaria treatment requires the use of good quality medication [1]. Poor quality medications may result in needless morbidity and mortality and can facilitate emergence of drug resistance [2]. Reports of various surveys from South East Asia showed that up to 50 % of the artesunate monotherapy sold was fake and the situation was envisaged to get worse in malaria endemic countries, with the implementation of the ACT, which is more expensive [3–6]. Low and middle-income malaria endemic countries are prone to a number of risk factors for poor quality ACT. Primarily these include ineffectual drug regulation and inadequate technical capacity, which are compounded by a lack of political will and resources [7].

Medicines quality is divided into four main classes; quality assured; falsified (counterfeit); substandard; or degraded. But there are no universally-accepted definitions of these categories [8]. Quality-assured medicines have the acceptable amount of active pharmaceutical ingredients as specified by the pharmacopeia’s and meet other quality attributes; falsified medicines do not contain the stated active pharmaceutical ingredient (SAPI) and may carry false representation of their source of identity. A falsified drug could signal a potential counterfeit product, which does not comply with intellectual property rights or may infringe trademark law [9]. Substandard drugs are produced with inadequate attention to good manufacturing practices and may have contents or dissolution times that are outside accepted limits, due to poor quality control [10, 11]. Degraded formulations may result from exposure of good-quality medicines to light, heat, and humidity. It can be difficult to distinguish degraded medicines from those that left the factory as substandard, but the distinction is important because the causes and remedies will be different.

## Purpose of the meeting

The ACT Consortium drug quality programme (ACTc-DQP) held a meeting in Geneva, Switzerland, and shared findings from their multi-country study that assessed the quality of over 10,000 artemisinin-based combinations, purchased in six malaria endemic countries. The meeting was attended by 34 representatives from 20 institutions, including the World Health Organization (WHO), United States Pharmacopeia (USP), the Global Fund (GF), INTERPOL as well as other academic institutions. Participants included policymakers, programme managers, researchers, technical advisors and donors.

David Schellenberg, director of the ACT Consortium, opened the meeting, citing earlier reports that as many as a third of anti-malarials are ‘fake’ [12–14]. Such reports formed the rationale for the ACT Consortium drug quality programme to investigate the quality of artemisinin-based combinations at a larger scale than in previous studies; using a representative sampling approach and standardized methodology to assess the prevalence of fake and substandard artemisinin-based combinations across a range of countries in sub-Saharan Africa. The aim was to provide Ministries of Health with the evidence upon which local regulators can take action.

## Public health impact: a focus on drug quality

The public health impact of poor quality ACT is stark. The focus is often on falsified drugs, and the direct dangers for the patient i.e. delayed clinical recovery and increased mortality. However, a further public health concern is the potential indirect impact, through the promulgation of drug resistance due to substandard anti-malarials.

To counter this threat requires routine surveillance systems and technical capacity to monitor drug quality on an ongoing basis, supported by effective regulatory action at national and international level. Souly Phanouvong, senior manager, Asia programmes, USP started the meeting and his presentation set the scene by outlining the technical, coordination, capacity and operational challenges they had encountered during monitoring drug quality in the Mekong sub-region. Contributing factors to problems with medicine quality were identified as weak institutional capacity that does not ensure good quality medicines are produced, procured, supplied and distributed to patients. There remains a need to strengthen surveillance, build capacity in country by ensuring that national quality control laboratoriess are sufficiently equipped to carry out the work. Once data is generated it needs to be shared in a timely manner which needs people with experience for the task. Added to the effort is the need to identify the key suppliers, manufacturers and distributors of poor quality drugs and have the regulatory capacity in place at the national level to root out the problem.

## Status of ACT quality

Establishing the scale of the problem of poor quality ACT remains a challenge. The WWARN Antimalarial Quality Surveyor Database (AQSD) was created to collate and present a comprehensive overview of the quality of anti-malarials, incorporating reports from both the scientific and grey literature [15]. It includes studies on anti-malarial drug quality spanning the past 67 years and indicates that around 30 % of anti-malarials tested have not met the criteria for good quality drugs, containing the acceptable amount of SAPI [16]. However, comparison of drug quality findings across time and place is hampered by methodological differences in the sampling strategies and laboratory techniques used in different studies. In some cases, it is not clear whether the samples examined were representative of all anti-malarial drugs on the market. Geographical disparities have also been found in the data available. This is especially the case in sub-Saharan Africa where the overview of anti-malarial drug quality is dominated by data from three countries—Nigeria, Tanzania and Ghana.

### Sampling strategies

An epidemiologist’s perspective was presented by Siȃn Clarke of the ACTc, on the challenge of assessing the extent of the ACT quality problem. She focused on sampling strategies to collect drugs, including their advantages and disadvantages (Table 1), illustrated by an analysis of the data points included in the AQSD during the last 5 years (2010–2015). The majority of reports, scientific studies and national drug quality surveys from Africa used a convenience sampling approach. This is a type of non-random sampling in which surveyors may sample drugs from outlets based primarily on ease of access or perceived risk [17]. Purposive and convenience approaches are efficient and cost-effective, but are more likely to be flawed by selection bias. Indeed, if the AQSD data points are compared according to the sampling method that was used, the proportion of medicines that fail testing was generally higher in studies that used convenience sampling than in studies that used random sampling. Random sampling should yield a more representative estimate of prevalence, provided the sampling frame (list of outlets) is comprehensive and up-to-date. The reliability and generalizability should be robust and the results can be replicated.

**Table 1:** Comparative strengths and weaknesses of three sampling approaches [18]

Sampling approach	Strengths	Limitations
Convenience	Rapid Low cost	Sampling depends on collectors choice of outlet (risk of bias) Poor documentation—findings hard to replicate Prevalence estimates are not reliable
Random	Sampling frame is defined to obtain representative sample from all types of outlets and/or brands Results can be replicated	Need to authenticate and update sampling frame increases time and cost of survey
Mystery clients	Outlets are unaware of survey so less chance of sample bias	Information on sources of poor quality drugs limited to brand, batch and country of manufacture as stated on packaging
Overt	Additional information is collected at minimal additional cost to mystery approach as provider is aware of the aims of the study	Risk of sampling bias in samples collected, if some outlets refuse to sell or samples are deliberately withheld as poor quality is known by the seller

At the outlet, the drug samples to be tested may be procured using a covert approach (so-called ‘mystery shopper’), where the researcher poses as a patient and asks for a drug to treat malaria. This has a relatively low risk of sampling bias, but the number of brands that can be obtained will be limited. An alternative approach is overt sampling in which the researcher informs the outlet staff of the purpose of the research and requests drugs, and completes a short questionnaire. There is a risk of sampling bias if outlets refuse to be sampled or are aware of which samples might be poor quality. The advantage is that more samples and additional information, for example regarding the supply chain or educational level of the provider, can be collected at minimal incremental cost. The results can thus provide more detail than the covert approach, such as sources of poor quality drugs, but may be compromised if sampling bias occurs.

Despite these considerations, results from the ACTc studies in Nigeria [18] and Cambodia [19] found that there was little difference between the drug samples obtained using overt and mystery approaches. However, artemisinin monotherapies (marketing for which is now prohibited by the WHO for uncomplicated *Plasmodium falciparum* malaria and subject to a national ban on sales in some countries) [20] were more readily detected through mystery clients than through overt sampling. This suggests that the reliability of the sampling approach used may also depend on the type of products sampled and the local regulatory context.

### Techniques for analysing drug quality

A well-equipped medicines quality control laboratory (MQCL) is the crucial component of any drug quality assurance system; with a range of analytical equipment such as high performance liquid chromatography and mass spectrometry systems, as well as quality-assured reference standards, all of which is cost intensive. An MQCL also requires staff with a high level of technical expertise and extensive knowledge of method development.

The need for the rapid detection of poor quality drugs through the supply chain has seen the development of hand held devices, based on spectroscopic methods, for use as screening tools. A broad overview of currently available technologies was presented by Ben Wilson of Intellectual Ventures Laboratory/Global Good. Recent advances in such technologies depend on being both cost-effective and easy to operate as they use a variety of approaches to assess drug quality including; product recognition, detection of SAPI and composition determination in the effort to detect the falsified drug.

Product recognition aims to establish whether the packaging is genuine or not. This requires a sample of the original manufacturer’s packaging or being familiar with key features of the packaging. The technologies being developed include handheld versions of methods previously used only in laboratories, such as mass spectrometry (MS) and nuclear magnetic resonance (NMR) [21]. Despite the accuracy of these technologies, they are complex to use (especially in a field setting), requiring specialist skills to operate and are cost intensive (around £50,000 per unit) [22].

Two main approaches to detect a SAPI or identify falsified medications were discussed; spectroscopy techniques and separation techniques. The former utilizes infrared spectroscopy e.g., the Raman handheld device [23], which involves acquiring spectra from a drug blister and comparing it with a library of spectra from well-characterized samples. The cost of infrared spectroscopy and performance is dependent on the wavelength range (broader the range cheaper the price and more defined ranges entail greater price). Devices provide purportedly fairly accurate results and are straightforward to use once the individual has been appropriately trained, however their capital cost (in the range of $2625–$17,485 for a Raman handheld device) may be prohibitive [19]. The cost per test is a relative advantage of spectroscopy methods. Separation techniques such as paper-based chromatography allow the simple testing of multiple SAPIs on a single piece of card (known as multiplexing) [20–22]. This is very simple to use and inexpensive but is poor at SAPI quantitation. Another separation technique Pharmacheck [23] uses photoluminescence and microfluidics in place of paper and colour change and purports to be quantitative thus enabling detection of substandard drugs. A rapid, semi-quantitative, simple to use and low-cost thin layer chromatography-based test that specifically detects the artemisinin component of ACT, has been developed at and patented by London School of Hygiene and Tropical Medicine (LSHTM) [24].

Despite a range of screening devices available there is as yet a lack of a systematic comparison in terms of their cost, performance and ultimately chemical content analysis, with methods listed in international pharmacopeia, such as high performance liquid chromatography (HPLC), which measures the quantitative amount of SAPI and is regarded as the gold standard [25].

### Findings from the ACT Consortium drug quality programme

**Fig. 1:**
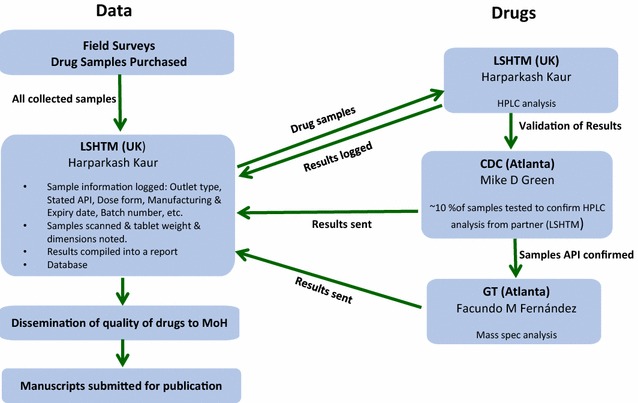
Diagram of sample flow and corroborative analyses at three independent laboratories

Reports of fake drugs formed the rationale for the ACTc-DQP to investigate the quality of ACT at a larger scale than in previous studies with the aim of providing Ministries of Health with evidence upon which local regulators could take action. Harparkash Kaur, the lead investigator of the ACTc-DQP presented the findings on behalf of the drug quality teams [11]. The ACTc-DQP collected 10,079 samples from ACT tablets from six countries; Equatorial Guinea (Bioko Island), Cambodia, Ghana, Nigeria, Rwanda and Tanzania, primarily using random sampling and both overt and mystery client approaches, Table 2. The samples were processed in the three corroborating laboratories as shown in Fig. 1. All sample information was logged onto the country specific databases. The samples from each study were analysed by HPLC at LSHTM, UK and a duplicate set was sent to Michael Green’s team at the Centre for Disease Control, Georgia, USA, who randomly selected ten percent of samples out of the total sent and analysed them using HPLC to corroborate the results from LSHTM. Additional validation was provided by Facundo Fernandez’s team, who analysed a duplicate set of samples using MS at the Georgia Institute of Technology, USA to confirm the presence of the stated SAPI or the detection of unstated compound instead. Laboratory analysis results for each sample were added to the database of sample information for each country.

**Table 2:** Numbers of samples analysed and quality of ACT found per country

Country (date of sampling)	Number of samples	Number of brands	Percent quality assured	Percent falsified	Percent Substandard	Artemisinin monotherapy tablets
Rwanda (2008)	97	1	93.8	Not found	6.2	Not found
Cambodia (2010)	291	21	68.7	Not found	31.3	Found
Ghana-Kintampo (2011)	257	31	63.0	Not found	37.0	Not found
Tanzania (2010)	1737	37	88.0	Not found	12.0	Found
Tanzania (2011)	2546	46	97.8	Not found	2.2	Found
Nigeria-Enugu Metropolis (2013)	3024	131	92.2	1.2	6.6	Found
Nigeria-Ilorin city (2013)	1450	77	91.5	0.8	7.7	Found
Equatorial Guinea-Bioko Island (2014)	677	142	91.0	7.4	1.6	Found

Results from the three laboratories enabled robust estimates of quality and cross-validation demonstrating that if the sampling strategy is representative and the analytical methods are of the highest standard, then findings are repeatable and reliable. These are important considerations when an issue is identified with drug quality and it becomes necessary to track the impact of efforts made to improve drug quality.

The findings from six countries show that the proportion of falsified drugs is much lower than the reported ‘one third’ in previous studies. The ACTc-DQP team tested less than the 20 tablets per sample as stipulated by USP, hence they used wider tolerance limits of 85–115 % to classify drugs to be of acceptable quality instead of the 90–110 % given by USP and WHO [26]. The wider tolerance limits may underestimate the proportion of substandard drugs but they will not affect the estimates of the falsified drugs found. Results from their study published the day before the dissemination meeting documented the quality of ACT from Enugu State, Nigeria [18]. Kaur reported that just 1.2 % of 3024 ACT samples collected from every known pharmacy, patent medicine vendor and public health facility, were falsified (did not contain the SAPIs). Furthermore two other published ACTc studies from Cambodia and Tanzania detected no falsified ACT [19, 27]. However, substandard ACT was found in each of the six countries, with as many as 31.3 % of 291 samples collected in Cambodia [19] failing to meet the tolerance limits (i.e. SAPIs between 85 and 115 %) for acceptable quality drugs. The findings demonstrate that the threat of substandard drugs is in some instances greater than that of falsified drugs and merit more attention than they have received so far.

The focus in recent years has been on counterfeit or falsified drugs, especially anti-malarials, however these results indicate that the risk of substandard drugs has perhaps been understated.

### Degraded products

The knowledge base for degraded drugs is sparse at best and non-existent for ACT, reported Harparkash Kaur. The ACTc-DQP, LSHTM team, also undertook additional studies to determine the stability of ACT tablets and degraded products.

‘Natural ageing’ of 2880 samples each of artemisinin-based combinations Coartem^®^ (artemether–lumefantrine) and ASAQ Winthrop^®^ (artesunate-amodiaquine) was undertaken to evaluate their long-term stability in tropical climates. Samples were aged in the presence and absence of light, on-site in Ghana and in a stability chamber (London), removed from each site at regular intervals and analysed to measure loss of the SAPIs over time and, detect products of degradation. Loss of SAPIs in samples (both in Ghana and the stability chamber), was 0–7 % over 3 years (~12 months beyond expiry) with low levels of degradation products detected [28]. In addition, none of the degradation products were found to exhibit anti-malarial activity. Presence of degradation products together with evidence of insufficient APIs can be used to classify drugs as degraded.

### Drug quality and the emergence of resistance

The impact of substandard drugs is particularly worrying when viewed in the context of drug resistance, specifically to artemisinin derivatives [29]. Philippe Guérin, Director of the World Wide Antimalarial Resistance Network [WWARN], reported that there are now confirmed cases of artemisinin resistance in Cambodia, Laos, Thailand and Myanmar. The mechanisms of resistance are complex and not yet fully understood. Poor quality ACT is one of many factors that are likely to fuel drug resistance, for instance if sub-therapeutic levels of SAPIs are included in ACT formulations. However the most important driver of artemisinin resistance may be the prolonged use (nearly 30 years) in South East Asia of artemisinin monotherapy. The gravest concern is the spread of resistance beyond South-East Asia and into sub-Saharan Africa, which could lead to a potentially cataclysmic situation, effectively wiping out a decade of investment in malaria control and treatment programmes [30].

*'Has everything been done to prevent or delay the evolution of drug resistance. Preventing the global spread is not an on-off button, its a complex mechanism. It requires us to recognise the science behind drug resistance, which must be translated into public health action. It can be achieved by improving the value of existing data, preserving efficacy of drugs in current use, detecting and managing resistance spread/emergence and ensuring efficacy of new drugs'.*

## Strengthening drug quality surveillance and regulation

Presentations by USP, WHO and Interpol reflected on progress with ongoing initiatives to strengthen the capacity of national and international networks to monitor drug quality. The USP Promoting the Quality of Medicines programme [31] in part seeks to strengthen regulatory, technical and drug quality monitoring capacity and is most advanced in parts of the Mekong region in South-East Asia. This has included setting up sentinel sites for drug quality monitoring in several countries as well guiding and assisting in the regular sampling of anti-tuberculosis, anti-retrovirals and anti-malarials. Working with governments and regulatory agencies has enabled the closing down of outlets, apportioning of fines, seizure of remaining stocks and blacklisting or delisting of outlets from registration, as punishments for selling or cooperating with those who sell counterfeit drugs. USP have also identified four areas that require urgent investment; education and advocacy, availability of drugs, affordability of quality-assured drugs and enabling leadership at government level.

*'Leadership at the government level is key, we are struggling in all 35 countries in which we work'.*

### Surveillance and monitoring of drug quality at the global level

As a major supplier of ACT to many malaria endemic countries ensuring the integrity of supply chains is an integral interest for Global Fund (GF). Andrew McLoughlin, Officer of Inspector General, at the GF, presented on the issues related to strengthening supply chains to prevent leakage (theft)/diversion of donor-funded anti-malarials. This leakage (theft)/diversion of quality assured donor-funded anti-malarials creates stock outs at public health facilities that result in patients being turned away without treatment. Stock-outs then force the financially able patients to seek anti-malarials from alternative sources, such as pharmacies and street markets, creating a demand whereby those vendors may unknowingly sell counterfeit anti-malarials. Most of the stolen drugs have been identified to be sold in pharmacies and street markets both in the country that the anti-malarials were delivered to and other countries.

The WHO has established the Members State Mechanism for the surveillance and monitoring of drug quality on an international scale, which was presented by Michael Deats (WHO). The Surveillance and Rapid Alert System for Substandard/Spurious/Falsely labelled/Falsified/Counterfeit (SSFFC) Medical Products [32] was devised by the WHO to tackle the threat of contaminated products circulating around the globe, a problem perpetuated as a result of increasing globalization. A number of countries lack technical capacity for conducting not just quality assurance, but more complex forensic analysis, issues that the WHO is well equipped to address by dispatching expert teams in response to urgent requests for assistance made by member states. The surveillance and monitoring system was established to better gauge the scope, scale and harm caused by SSFFC medical products and provide technical support and alerts where required. In addition, a well-functioning surveillance system produces a validated evidence base for policy makers. Since 2013, the programme has engaged 113 member states with over 920 suspect products reported from 83 countries. Falsified drugs remain a concern with WHO receiving regular reports i.e. 126 of suspected falsified artemether/lumefantrine (innovator and generic versions), from 14 sub-Saharan African countries have been filed since July 2013. On investigation by the WHO, they were found to have less than 10 genuine medicines. A database logging reports of SSFFC and substandard drugs has been created and with relevance to falsified anti-malarials, 57 % of those reported so far have been artemisinin-based.

The types of reports and their source vary from country to country. Some are raised by national drug regulatory or medicines control agencies and others originate from an individual healthcare facility or provider. The most detailed reports are initiated by patients or health professionals however these make a small proportion of the total number. This highlights the need to embed the reporting of suspect products into national regulatory systems and data sharing.

### Building capacity at the regional and national level

Both INTERPOL and WHO identified the importance of the formation of collaborative regional networks that shared information on the detection of a SSFFC or substandard drug. Such networks enable triangulation of the source of the suspect product. Subsequent action against either the distributor or the manufacturer can then be taken. However, many countries do not have the judicial powers or capability to act. Aline Plançon from the pharmaceutical crime programme within INTERPOL stated that in these instances they can assist, issuing international arrest warrants for unscrupulous manufacturers based in a different country to the one in which the product was discovered. In the last five years, there has been a noticeable rise in the number of warrants issued and subsequent criminal cases for counterfeiting. However complexities arise given the international nature of some of the major cases.

*'Having a law is one thing, having a criminal justice system that can manage and enforce that is something different'.*

At a national level there is a need for better coordination between and across a myriad of agencies including health and drug regulatory bodies, customs and the police, underpinned by appropriate and enforceable legislation. This requires political will and government engagement without which a national regulator’s authority to take action against distributors or producers of SSFFC and substandard drugs is futile.

*'The countries that do best have multi-sectorial stakeholder engagement, regulatory, police, customs, the private sector and not just manufacturers, but importers and retailers as well. In some parts of the world it’s a legal requirement that these are linked together. One step in prevention is encouraging lots of regular interaction among these groups. That translates into practical, on the ground benefits'.*

## Further considerations

An open discussion at the end of the meeting elicited some essential additional considerations.

### Prioritizing prevention

According to the WHO, INTERPOL and USP, systems exist for detecting SSFFC and medical drugs. These systems track, respond to and address to some extent the threat of such drugs on a national, regional and international level. However it was agreed that prevention must become a priority. The aforementioned multi-sectoral approach is imperative to preventing the proliferation of poor quality drugs in terms of substandard which will need a different approach to tackle than counterfeit or falsified drugs. Causes and people involved will vary. Engaging with Ministries of Health, empowering regulatory agencies and forging close ties with the private sector would vastly reduce the risk of such drugs circulating in a country.

Furthermore, on both a national and international scale pharmaceutical industries must become actively involved to curb the rise in SSFFC and substandard drugs. This includes being more transparent about their own drug quality data and engaging with stakeholders to build capacity.

*'Transparency from industry about their own reports of drug quality is required as usually this kind of information is understandably kept confidential. However, more transparency would lessen the concerns that industry is hiding information. More transparency will allow for more collaboration'*.

### Improving collaboration and coordination

Effective regulatory action is dependent on national, international and regional collaborations including timely communication, data sharing, standardized definitions and methods. A multitude of drug quality screening technologies exist. There was a consensus that better collaboration is required in the developing and scaling up of these technologies. The GPHF Minilab^®^ [33] remains a key component of drug quality surveillance systems. Currently many in-country laboratories have trialled alternative technologies but few are actually being utilized. Concerted effort amongst pharma and academic institutions is likely to produce technologies suitable for screening drugs in the field.

The multi-disciplinarily profile of the participants was also emphasized, which may potentially complicate coordination and subsequently result in duplication of work. However, the range of disciplines must be allowed to figure more prominently to enable the formulation of a broader picture of the issue of drug quality. There is a requirement to shift the focus of drug quality from the technical paradigm toward a holistic model; involving providers, industry and regulators.

*'There is scope to develop multidisciplinary approaches to gain a fuller picture: chemical content, epidemiology, statistics, economics, anthropology, etc.'*

Finally the need for better dialogue with the private sector was discussed. In low-income settings in particular, the private sector and more specifically the informal sector are at greatest potential risk for poor quality drugs. In some instances, these vendors are unaware they are selling such drugs and prosecuting them for doing so may not be the best approach to tackling the problem. The GF in collaboration with other donors and the WHO work to assure the integrity of the supply chains to reduce (stop) the demand resulting from the leakage (theft) of quality assured anti-malarials. Andrew McLaughlin and Aline Plançon also mentioned that the detail of their operations cannot be revealed in an open forum.

Discussions have since resulted in USP and WHO to embark on joint initiatives of training workshops, to produce specific training module on sampling procedures as well as surveillance and monitoring.

## Conclusions and recommendations

A major premise of the ACTc drug quality project has been to establish the facts relating to the prevalence of ACT quality in selected countries. The systematic, and rigorous approaches to sampling and drug quality analysis reduced the risk of bias and produced considerable data that shows poor quality drugs not to be as prevalent as previously reported. Whilst the numbers of falsified drugs were either none or less than 8 % but the substandard drugs were found between 6.0 and 37 % in all countries where studies were conducted. In the selected countries, the ACTc provided a clear snapshot of the status of the quality of ACT at the time of the surveys. However sustainable systems need to be developed to enable ongoing monitoring of drug quality at country level. A combination of approaches and methodology to sampling is likely to be necessary. Given the ease and affordability of convenience sampling this is likely to remain an important approach to detecting the presence of poor quality drugs in a market. Rapid field tests may be useful for screening of such samples. Detection of a problem should trigger a representative sampling of drugs so that unbiased estimates of the scale of the problem can be generated. Quality-assured laboratory analyses of samples is essential, but the development of the necessary capacity in every country is a long-term ambition. In the meantime, the model developed by USP in south-east Asia, where laboratories are designated at different levels of capability with regional reference laboratories is gradually being replicated in Africa.

Future drug quality studies should, where feasible, employ systematic approaches to sampling and analysis and a updating of the MEDQUARG guidelines (a checklist of items that should be included in reports of medicine quality) maybe required [17]. Finally on a local, national, regional and international level stakeholders from various disciplines with an interest in drug quality must work in tandem to advocate for more attention to be focused on poor quality drugs. This is most crucial at a national level where leadership and political will are two key drivers in building regulatory and technical capacity (testing the medicines in country to inform the regulator).

The key recommendations emerging from this meeting are:

- A need for more systematic approaches to sampling and testing of drugs
- A more holistic and multidisciplinary approach to drug quality research requires incorporating anthropology, epidemiology, statistics and economics
- To encourage multi-stakeholder engagement on a national (and global) scale to include regulatory agencies, customs and police supported by tangible and enforceable legislation
- The need to invest and build national regulatory and technical capacity that is sustainable
- To focus towards prevention of poor quality drugs whilst maintaining surveillance and monitoring activities
- Develop a deeper understanding of the public health impact of substandard drugs both in terms of the immediate clinical implications as well as the propagation of drug resistance
- A shift in focus to the prevalence of substandard drugs whilst maintaining surveillance and monitoring of SSFFC including substandard drugs.

Falsified ACT remains a concern, but the results of these studies show that there is a need to increase the focus on substandard ACT, which will lead to drug resistance as a result of under dosing. The risk of poor quality ACT and falsified drugs is real and will continue to exist whilst unscrupulous or negligent manufacturers and distributors continue to operate. Co-operation and co-ordinated action will be required to stop the scourge of poor quality medicines.
